# Integration of proteomics with CT-based qualitative and radiomic features in high-grade serous ovarian cancer patients: an exploratory analysis

**DOI:** 10.1007/s00330-020-06755-3

**Published:** 2020-04-06

**Authors:** Lucian Beer, Hilal Sahin, Nicholas W. Bateman, Ivana Blazic, Hebert Alberto Vargas, Harini Veeraraghavan, Justin Kirby, Brenda Fevrier-Sullivan, John B. Freymann, C. Carl Jaffe, James Brenton, Maura Miccó, Stephanie Nougaret, Kathleen M. Darcy, G. Larry Maxwell, Thomas P. Conrads, Erich Huang, Evis Sala

**Affiliations:** 1 Department of Radiology, Cancer Research UK Cambridge Center, Cambridge, CB2 0QQ UK; 2 Department of Obstetrics and Gynecology, Gynecologic Cancer Center of Excellence, Walter Reed National Military Medical Center, Uniformed Services University, 8901 Wisconsin Avenue, Bethesda, MD 20889 USA; 3 The John P. Murtha Cancer Center, Walter Reed National Military Medical Center, Uniformed Services University, 8901 Wisconsin Avenue, Bethesda, MD 20889 USA; 4 Department of Radiology, Clinical Hospital Center Zemun, Vukova 9, Belgrade, 11080 Serbia; 5 Department of Radiology, Memorial Sloan Kettering Cancer Center, 1275 York Avenue, New York, NY 10065 USA; 6 Department of Medical Physics, Memorial Sloan Kettering Cancer Center, 1275 York Avenue, New York, NY 10065 USA; 7 Cancer Imaging Informatics Lab, Frederick National Laboratory for Cancer Research, Frederick, MD USA; 8 Department of Radiology, Boston University School of Medicine, Boston, MA 02118 USA; 9 Cancer Research UK Cambridge Institute, University of Cambridge, Li Ka Shing Centre, Cambridge, Cambridgeshire UK; 10 Cancer Research UK Cambridge Centre, Cambridge, Cambridgeshire UK; 11 Dipartimento Diagnostica per Immagini, Radiologia Diagnostica e Interventistica Generale, Area Diagnostica per Immagini, Radioterapia Oncologica ed Ematologia, Rome, Italy; 12 Department of Radiology, Montpellier Cancer Institute, INSERM, University of Montpellier, Montpellier, France; 13 Department of Obstetrics and Gynecology, Inova Fairfax Medical Campus, 3300 Gallows Rd., Falls Church, VA 22042 USA; 14 Inova Center for Personalized Health, Inova Schar Cancer Institute, 3300 Gallows Rd., Falls Church, VA 22042 USA; 15 Biometric Research Program, Division of Cancer Treatment and Diagnosis, National Cancer Institute, NIH, Rockville, MD 20850 USA; 16 Department of Radiology, University of Cambridge, Box 218, Cambridge Biomedical Campus, Cambridge, CB2 0QQ UK

**Keywords:** Ovarian neoplasms, Radiomics, Proteomics, Gene expression profiling, Prognosis

## Abstract

**Objectives:**

To investigate the association between CT imaging traits and texture metrics with proteomic data in patients with high-grade serous ovarian cancer (HGSOC).

**Methods:**

This retrospective, hypothesis-generating study included 20 patients with HGSOC prior to primary cytoreductive surgery. Two readers independently assessed the contrast-enhanced computed tomography (CT) images and extracted 33 imaging traits, with a third reader adjudicating in the event of a disagreement. In addition, all sites of suspected HGSOC were manually segmented texture features which were computed from each tumor site. Three texture features that represented intra- and inter-site tumor heterogeneity were used for analysis. An integrated analysis of transcriptomic and proteomic data identified proteins with conserved expression between primary tumor sites and metastasis. Correlations between protein abundance and various CT imaging traits and texture features were assessed using the Kendall tau rank correlation coefficient and the Mann-Whitney *U* test, whereas the area under the receiver operating characteristic curve (AUC) was reported as a metric of the strength and the direction of the association. *P* values < 0.05 were considered significant.

**Results:**

Four proteins were associated with CT-based imaging traits, with the strongest correlation observed between the CRIP2 protein and disease in the mesentery (*p* < 0.001, AUC = 0.05). The abundance of three proteins was associated with texture features that represented intra-and inter-site tumor heterogeneity, with the strongest negative correlation between the CKB protein and cluster dissimilarity (*p* = 0.047, *τ* = 0.326).

**Conclusion:**

This study provides the first insights into the potential associations between standard-of-care CT imaging traits and texture measures of intra- and inter-site heterogeneity, and the abundance of several proteins.

**Key Points:**

*• CT-based texture features of intra- and inter-site tumor heterogeneity correlate with the abundance of several proteins in patients with HGSOC.*

*• CT imaging traits correlate with protein abundance in patients with HGSOC.*

**Electronic supplementary material:**

The online version of this article (10.1007/s00330-020-06755-3) contains supplementary material, which is available to authorized users.

## Introduction

It is estimated that over 14,000 women will die from epithelial ovarian cancer in 2019 in the USA, the preponderance of whom will have high-grade serous ovarian cancer (HGSOC) [1]. Despite improvements in the treatment of ovarian cancer, the rate of recurrence remains high and both disease-free survival and overall survival are poor, due to resistance to standard platinum-based chemotherapy [2]. Although several clinical-pathological factors, such as age, stage, histologic grade, and surgical debulking status, have been shown to be important prognostic indicators of outcome in ovarian cancer patients, there are still no reliable biomarkers for predicting response to therapy and outcome [3].

Several studies have identified genetic markers that are associated with outcome in HGSOC patients [4–7], with TP53 the major driver mutation [8, 9]. Risk stratification using gene expression is reliable only in a subset of patients [6], as gene function may not necessarily correlate with the cognate gene product (e.g., protein) function. An analysis of 412 cases from The Cancer Genome Atlas (TCGA) study, which used reverse-phase protein arrays, identified a protein-driven index of ovarian carcinoma (PROVAR), which enabled classification of patients with different risks of recurrence and survival [10]. In addition, the Clinical Proteomic Tumour Analysis Consortium performed a mass spectrometry–based proteomic analysis of 174 ovarian tumors previously analyzed by the TCGA and integrated those data with genome-level data from a whole-exome sequencing [11]. This work showed that an abundance of selected proteins was associated with genomic changes, such as chromosomal structural abnormalities, copy number alterations, and the homologous recombination deficiency status. In addition, protein signatures were identified as a strong independent predictor for patient survival.

Imaging by computed tomography (CT) and, in selected cases, by magnetic resonance imaging (MRI), is used to evaluate the extent of disease and monitor treatment response in patients with HGSOC. To date, a few studies have applied radiomic feature analysis in patients with ovarian cancer. They showed that MRI-derived radiomic features can discriminate between benign and malignant ovarian masses, with a high accuracy of 87% [12]. Furthermore, CT radiomic features of patients with ovarian cancer correlate with response to therapy [13], progression-free survival [14, 15], and overall survival [15], and can identify patients at higher risk for recurrence [16]. Recent work by our group focused on evaluating the possible associations between CT imaging traits and texture metrics with genomics data and patient outcome [17–19]. The integration of clinical, proteomic, and radiomic data may enable to stratify patients according to risk for progression thereby allowing for tailored therapy [20].

In this pilot, hypothesis-generating study, we investigated the association between CT imaging traits and texture metrics with proteomic data in a small cohort of patients with HGSOC.

## Materials and methods

### Study population

This was a multi-institutional, institutional review board–approved, and Health Insurance Portability and Accountability Act (HIPAA)–compliant retrospective study, with waiver of informed consent from all institutions that participated in The Cancer Genome Atlas-Ovarian Cancer (TCGA-OV) Imaging Research Group [21].

**Fig. 1:**
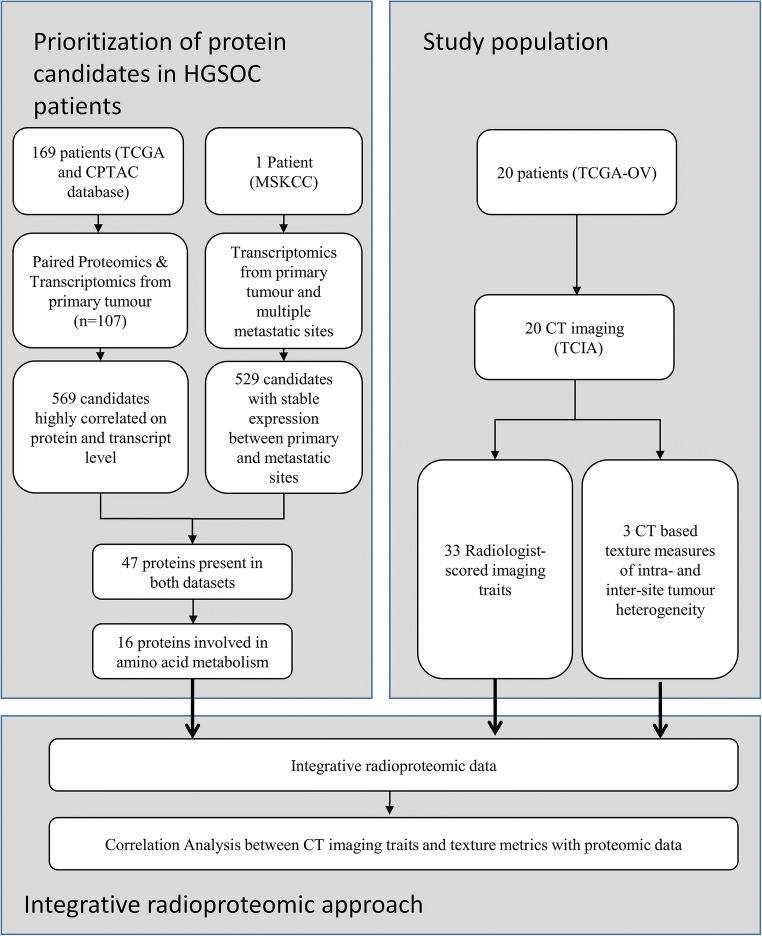
Study flow chart. Patients analyzed in this study were part of the TCGA dataset (*n* = 169). Protein candidates (5504 total) that exhibited stable expression between primary and metastatic sites and those that highly correlated with transcript abundance (*n* = 107 HGSOC patients, TCGA, and CPTAC) were selected using the Student’s *t* test (*p* value < 0.1) after boxCox normalization of transcript expression coefficients of variation and Spearman correlation distributions, respectively. Forty-seven genes exhibited both low correlation of variables between primary and metastatic sites (529 candidates) and were highly correlated at the protein and transcript levels (569 candidates). Among those, 16 proteins were associated with amino acid metabolism and selected for final analysis. CT data were available in 20 patients. Thirty-three radiologist-scored imaging traits and three CT-based texture measures of inter-site tumor heterogeneity were assessed and their correlation with the 16 cancer proteins was calculated

**Fig. 2:**
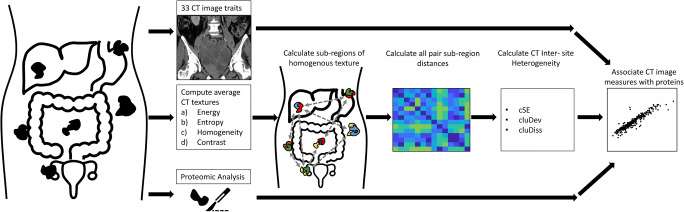
Overview of the experimental workflow. For each patient, 33 imaging traits, proteomics from a single-site tumor biopsy, as well as intra- and inter-site tumor heterogeneity texture measures, were obtained. The 33 imaging traits and measures of intra- and inter-site heterogeneity were correlated with proteomic data

The eligibility criteria included the following: (1) confirmed diagnosis of HGSOC, (2) HGSOC tissue submitted and analyzed by TCGA, (3) no neoadjuvant chemotherapy administered, (4) intravenous contrast-enhanced CT of the abdomen and pelvis performed prior to primary cytoreductive surgery, and (5) protein relative abundance measurements available. The final patient cohort consisted of 20 patients. Figures 1 and 2 summarize the experimental design. All patients were included in two prior studies that evaluated the association between the Classification of Ovarian Cancer (CLOVAR) subtype signatures and CT features in a single- [17] and multi-institutional setting [21], respectively. All patients were also included in two other studies that evaluated the feasibility of CT-based texture measures in the quantification of inter-site tumor heterogeneity and combined intra- and inter-site tumor heterogeneity [17, 22] in patients with HGSOC. None of these prior studies involved any analysis of protein abundance data.

### Image analysis

Thirty-six image features, including the 33 radiologist-scored CT imaging traits used in the study by Vargas et al, such as lesion size and laterality, locations of peritoneal disease, nodal stations involved, and locations of metastases, and three computer-extracted texture metrics were obtained [21]. All CT-derived variables are shown in Table 1 and Table 2.

**Table 1:** The CT-based image features used in this study, with possible values, summary statistics and inter-reader agreement

Feature	Possible values and summary statistics	Inter-reader agreement (%); Cohen’s kappa
Disease in mesentery	Yes: 50% (10/20) No: 50% (10/20)	95%; 0.90
Peritoneal disease in paracolic gutters	Yes: 80% (16/20) No: 20% (4/20)	100%; 1.0
Peritoneal disease in Pouch of Douglas	Yes: 75% (15/20) No: 25% (5/20)	85%; 0.57
Peritoneal disease in spleen/left upper quadrant	Yes: 85% (17/20) No: 15% (3/20)	100%; 1.0
Peritoneal disease in lesser sac	Yes: 30% (6/20) No: 70% (14/20)	90%; 0.76
Peritoneal disease in liver/right upper quadrant	Yes: 70% (14/20) No: 30% (6/20)	100%; 1.0
Number of locations with peritoneal disease	0: 10% (2/20) 1: 5% (1/20) 2: 5% (1/20) 3: 5% (1/20) 4: 30% (6/20) 5: 20% (4/20) 6: 25% (5/20)	90%; 0.62
Peritoneal disease calcifications	Yes: 5% (1/20) No: 95% (19/20)	95%; N/A
Peritoneal disease omental implant	Yes: 95% (19/20) No: 5% (1/20)	90%; 0.5
Shape of peritoneal disease	Diffuse: 15% (3/20) Predominantly diffuse: 50% (10/20) Predominantly nodular: 30% (6/20) Nodular: 0% (0/20) Peritoneal enhancement only: 0% (0/20) No peritoneal disease: 5% (1/20)	90%; 0.71
Infrarenal retroperitoneal lymphadenopathy	Yes: 30% (6/20) No: 70% (14/20)	100%; 1.0
Pelvic lymphadenopathy	Yes: 15% (3/20) No: 85% (17/20)	95%; 0.77
Inguinal lymphadenopathy	Yes: 0% (0/20) No: 100% (20/20)	100%; N/A
Porta/celiac/gastro lymphadenopathy	Yes: 25% (5/20) No: 75% (15/20)	100%; 1.0
Retrocrural lymphadenopathy	Yes: 5% (1/20) No: 95% (19/20)	95%; N/A
Supradiaphragmatic lymphadenopathy	Yes: 45% (9/20) No: 55% (11/20)	100%; 1.0
Thoracic lymphadenopathy	Yes: 0% (0/20) No: 100% (20/20)	100%; N/A
Number of locations with lymphadenopathy	0: 45% (9/20) 1: 15% (3/20) 2: 20% (4/20) 3: 5% (1/20) 4: 15% (3/20)	100%; 1.0
Metastases in liver	Yes: 0% (0/20) No: 100% (20/20)	100%; N/A
Metastases in lung	Yes: 5% (1/20) No: 95% (19/20)	100%; 1.0
Metastases in pleura	Yes: 10% (2/20) No: 90% (18/20)	100%; 1.0
Metastases in spleen	Yes: 0% (0/20) No: 100% (20/20)	100%; N/A
Metastases in other locations	Yes: 0% (0/20) No: 100% (20/20)	100%; N/A
Number of locations with metastases	0: 85% (17/20) 1: 15% (3/20)	100%; 1.0
Metastases calcifications	Yes: 0% (0/20) No: 100% (20/20)	100%; N/A
Size of pleural effusions	No pleural effusions: 65% (13/20) Small: 20% (4/20) Moderate/Large: 15% (3/20)	95%; 0.91
Pleural metastases removed	Pleural effusion: 5% (1/20) [NA]: 95% (19/20)	100%; 1.0
Pleural effusion metastases removed	Small: 5% (1/20) [NA]: 95% (19/20)	100%; 1.0
Size of ascites	No ascites: 15% (3/20) Trace or small: 40% (8/20) Moderate or large: 35% (9/20)	95%; 0.92
Mass laterality	No mass: 10% (2/20) Left: 5% (1/20) Right: 5% (1/20) Bilateral: 85% (16/20)	90%; 0.73
Mass calcifications	Yes: 10% (2/20) No: 90% (18/20)	95%; 0.64
Mass septations	Yes: 10% (2/20) No: 90% (18/20)	90%; N/A
Mass internal architecture	Cystic: 0% (0/20) Predominantly cystic: 0% (0/20) Mixed: 35% (7/20) Predominantly solid: 55% (11/20) Solid: 10% (2/20)	75%; 0.38
Length of largest lesion	Median: 63 mm Range: 24 mm to 116 mm	N/A

*N/A*, not applicable. Inter-reader agreement is shown as absolute agreement and Cohen’s kappa statistics for the two radiologists. In case of disagreement, a third radiologist acted as arbitrator. In case one or both readers scored all cases the same value, we were not able to calculate kappa values

**Table 2:** The texture features used in this study, with summary statistics

Feature	Distribution
Cluster site entropy	Median: 3.724 Range: 2.170 to 4.969
Cluster standard deviation	Median: 2.271 Range: 0.241 to 13.32
Cluster dissimilarity	Median: 4394 Range: 483.8 to 15,857

The CT images were a part of the TCIA TCGA-OV [23] collection in The Cancer Imaging Archive (https://cancerimagingarchive.net) [24], an NCI-supported archive of anonymized medical images. The cases were reviewed on a cloud-based virtual machine using the ClearCanvas with Annotation and Imaging Markup viewing software (Northwestern University) [25]. Image data in DICOM format, and features and segmentations generated for this paper, are available at 10.7937/TCIA.2019.9stoinf1 [26].

### CT-based qualitative imaging features

The CT imaging traits were assessed by two radiologists (E.S. and H.A.V.; 17 and 12 years’ experience in ovarian cancer imaging) independently, who were blinded to the patients’ clinical, pathological, and proteomic data. The details of the CT imaging trait evaluation are provided in the study by Vargas et al [17]. Briefly, each reader individually recorded the imaging interpretation on an electronic case report form, which was uploaded to a central server, compiled, and submitted for statistical analysis. Both ovarian masses and peritoneal disease were evaluated. If a definable ovarian mass was present, its features were recorded, including laterality, maximum size, internal architecture, and presence of calcifications and/or septations. The presence or absence of definable peritoneal implants and their specific location (small bowel mesentery, omentum, paracolic gutters, Pouch of Douglas, perisplenic/left upper quadrant region, lesser sac, and perihepatic/right upper quadrant region) was also recorded. Lymphadenopathy was defined as a short-axis dimension above a predefined size cutoff specific to each location or specific imaging appearance, as detailed in Vargas et al [21].

In the event, the two radiologists disagreed on a categorical feature (e.g., presence of peritoneal disease in the mesentery), and a third radiologist (L.B. 5th year of training) served as an arbitrator. For quantitative features, such as lesion size, the measurements of the two radiologist were averaged. The number of sites in which peritoneal disease was present was also recorded and was considered an additional human-read feature. This was also done for the presence of lymphadenopathy and intra-parenchymal metastases.

### CT-based texture analysis

A detailed description of image segmentation and texture feature extraction are provided in Veeraraghavan et al [22]. A slice thickness of 5 mm was used for analysis. kVp were variable, as CT machines from different vendors in different institutions were used. In brief, two oncologic imaging research fellows with 4 and 6 years of experience (M.M., S.N.), respectively, in consensus, manually segmented primary ovarian tumor(s) and all metastatic tumor implants in the abdomen and pelvis. Segmentation was performed using 3DSlicer [27] by tracing the contour of each lesion on each slice to produce the volumes of interest (VOI). In total, tumor burden in 14 distinct anatomical regions was manually segmented. Voxel-wise Haralick textures (energy, entropy, contrast, and homogeneity) were computed from within the manually delineated VOIs using in-house software implemented in C++ using the Insight ToolKit [28]. Site-specific sub-regions were computed by voxel-wise clustering of the Haralick textures using the kernel K-means method [29]. Following clustering, tumor sites were divided into distinct sub-regions of similar texture features. The intra- and inter-site heterogeneity (IISTH) measures (i.e., cluster site entropy, cluster standard deviation, and cluster dissimilarity) were computed. IISTH measures summarized the heterogeneity across all tumor sites in each patient. The more different each site of disease, the higher are the IISTH measures.

### Protein abundance measurements

Given that the molecular profiles of TCGA patients were based on primary ovarian tumor specimens and our radiologic measurements were made on metastatic patterns of disease, we sought to identify proteins conserved between primary and metastatic tumors in an ovarian cancer patient and then cross-reference these with proteins associated with radiologic patterns of metastasis and proteomic profiles in TCGA. Protein relative abundance measurements from the CPTAC analysis of 107 TCGA HGSOC patients were available for 3377 proteins (normalized log-ratios, standardized across two institutions), which were obtained from Zhang et al [11]. When multiple measurements were available, their abundances were averaged.

As we included only 20 patients in this study, a simple correlation analysis between the protein abundance of 3377 proteins and the CT imaging traits and texture features would result in a high number of false-positive results. Therefore, the purpose of the workflow described below was to reduce the number of proteins used for the final analysis. First, the 3377 proteins were prioritized for inclusion in the analysis, based on evidence of conserved transcript expression between primary and metastatic sites and on agreement between transcript and protein abundance levels. Transcript expression (Affymetrix) data (Supplemental Table 2 in reference [30]) from the primary tumor and multiple metastatic sites (spleen, right-upper-quadrant, liver, and vaginal cuff) from an HGSOC patient [30], along with transcript expression (mRNA-seq) data from primary HGSOC tumors (*n* = 107) [31] with matching protein expression data from the same case set (*n* = 107) [11], were merged by gene name, yielding a final matrix of 5504 co-measured proteins and transcripts (Supplemental Table 1, in reference [11]). Second, coefficients of variation (CV) values in transcript expression between the primary and metastatic sites, as reported by Jimenez-Sanchez et al [30], and Spearman correlations calculated for transcript and protein expression from the *n* = 107 patients, were normalized using the boxCox function in R (version 3.3.2). Transcripts that exhibited a low CV between the primary and metastatic sites (i.e., stable expression independent of metastatic loci), and with expression levels highly correlated to their proteins, were selected by comparison with normal distributions using *T* test analyses and a significance threshold of a *p* value < 0.1. These analyses identified 529 candidates with conserved expression between primary and metastatic sites and 569 candidates highly correlated at the protein and transcript level; 47 of these intersected between these two analyses. To further reduce the number of proteins, we prioritized proteins based on their molecular function and their interaction with each other using MetScape [32]. Sixteen proteins were associated with the regulation of the amino acid metabolism (*p* < 0.001). The selection of these 16 proteins was done solely on the basis their summary statistics (coefficients of variation in transcript expression between primary and metastatic sites, correlations between transcript and protein expression) and molecular function while correlations of imaging features were not considered in any way in selecting these proteins. Finally, these 16 candidates were included in subsequent analyses to identify those that further correlated with clinical imaging features (Fig. 1). The 16 proteins are given in Supplementary Table 1.

### Statistical methods

The percentage agreement and the Cohen’s kappa coefficient used to assess the agreement between the two radiologists were calculated for all CT-based imaging features. Univariate associations were assessed between the expression levels of the proteins and CT-based image features in which previous results provided evidence of prognostic power (presence of peritoneal disease in the mesentery and diffuse—non-mass forming—peritoneal involvement, as found in the study by Vargas et al [21]: number of sites with peritoneal disease; presence of peritoneal disease in the liver; presence of Pouch of Douglas implants; presence of supradiaphragmatic lymphadenopathy; and non-visualization of a discrete ovarian mass). For binary image features, these associations were assessed using a Mann-Whitney *U* test to compare the expression levels of a specific protein among patients with the feature to those among patients without the feature. The area under the receiver operating characteristic curve (AUC) was reported as a metric of the strength and direction of this association. For quantitative features, these associations were assessed through inferences on the Kendall tau rank correlation coefficient.

Univariate associations between the texture radiomic features (cluster site entropy, cluster standard deviation, and cluster dissimilarity) and protein relative abundance were assessed through inferences on the Kendall tau rank correlation coefficient. The Benjamini-Hochberg procedure was used to correct for multiple hypothesis testing. All tests were performed at the *α* = 0.05 level.

## Results

### Patient characteristics and distribution of disease

**Fig. 3:**
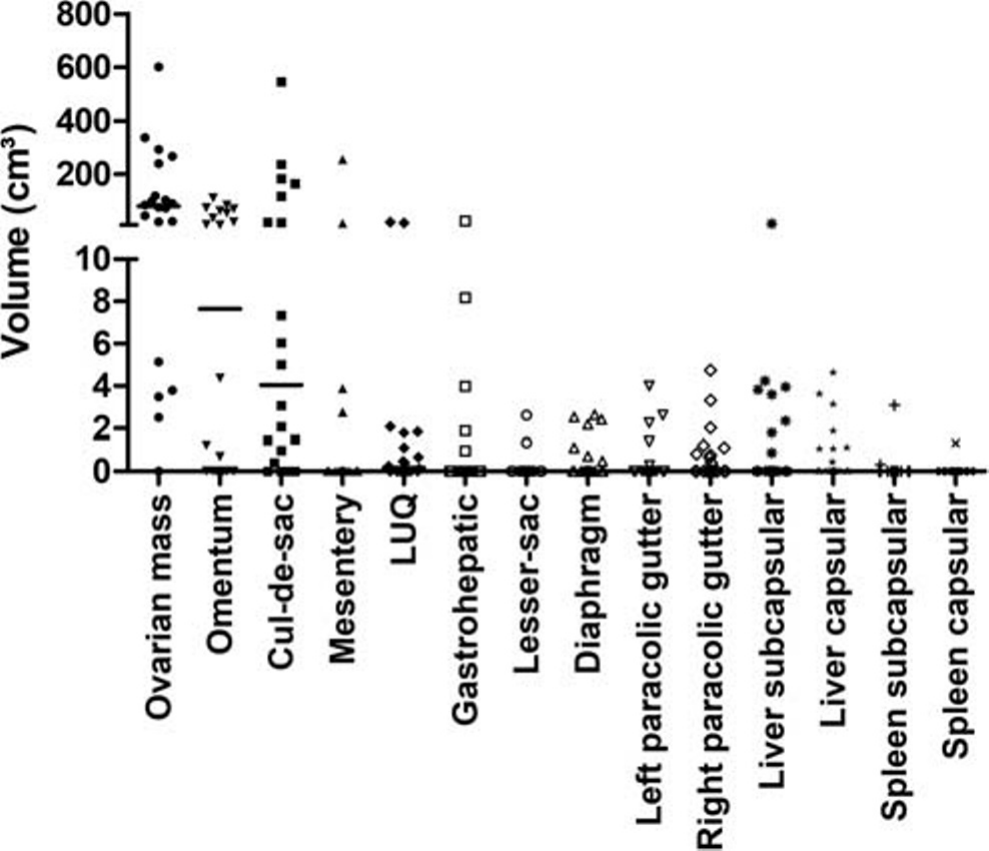
Tumor volume of the sub-regions of the 20 patients included in the final analysis is shown. LUQ, left upper quadrant: the horizontal line indicates the median value

The patients’ characteristics are shown in Table 3. The median number of tumor sites was six (range, 1–11). The median tumor volume was 192 cm^3^ (range, 12–2574 cm^3^), the highest being the tumor volume of the ovarian masses, with a median of 80 cm^3^ (range, 0–600 cm^3^). Figure 3 illustrates the tumor volumes for each anatomical sub-region.

**Table 3:** Clinical characteristics of the 20 patients used in the analysis

Characteristic	
Median age (year)	60.5 (49–80)
FIGO stage III	8 (40%)
FIGO stage IV	12 (60%)
Residual disease following primary debulking	
None	5 (25%)
Less than 1 cm	11 (55%)
Greater than 1 cm	3 (15%)
No. of patients with missing data	1 (5%)
Median TTP (day)	426 (9–1475)
Median OS	1469 (9–2749)

Age, TTP, and OS are shown as median (range), while the remaining variables are shown as number of patients; *TTP*, time to progression; *OS*, overall survival

### Inter-reader agreement of CT-based imaging traits

There was a good to excellent inter-reader agreement for CT-based imaging traits, with an absolute agreement ranging between 75% (κ = 0.32) for the variable mass internal architecture to 100% (κ = 1.0) agreement for the majority of variables (Table 1).

### CT imaging traits are associated with an abundance of several proteins

Four CT imaging traits were associated with an abundance of several proteins (Table 4). For example, disease in the mesentery was associated with reduced levels of CRIP2 (*p* = 0.0002, AUC = 0.05) and GPI (*p* = 0.004, AUC = 0.22). Supradiaphragmatic lymphadenopathy was associated with decreased abundance of ALDH3A2 (*p* = 0.02, AUC 0.192) and increased abundance of MAGEA4 (*p* = 0.046, AUC = 0.768). The number of sites with peritoneal disease was correlated with increased levels of ALDH3A2 (*p* = 0.03, τ = 0.36) and CRIP2 (*p* = 0.01, τ = 0.378). The shape of peritoneal disease was associated with the abundance of MAGEA4 (= 0.04, τ = − 0.343). After correction for multiple testing through the Benjamini-Hochberg procedure, the correlation between CRIP2 and the presence of supradiaphragmatic lymphadenopathy remained significant (corrected *p* = 0.03). Patients with without or diffuse peritoneal disease had higher levels of MAGEA4 (median 2.31 and 2.31 respectively) than patients with predominantly diffuse or predominantly nodular disease (median − 0.233 and − 1.51 respectively).

**Table 4:** Associations between protein abundance and CT-based imaging traits previously found to be prognostic in patients with HGSOC. Only associations for which the *p* value was less than 0.05 were included in this table. AUC values greater than 0.5 indicate a positive association between the imaging trait and the level of expression of the protein whereas AUC values less than 0.5 indicate a negative association. Positive values of *τ* indicate positive association between the imaging trait and the level of expression of the protein whereas negative values of indicate negative association

Image trait	Protein	*p* value	Corr. *p* value	Point estimate of metric of association
Disease in mesentery	CRIP2	0.0002	0.03	AUC = 0.05
	GPI	0.04	0.51	AUC = 0.22
Supradiaphragmatic lymphadenopathy	ALDH3A2	0.02	0.45	AUC = 0.192
	MAGEA4	0.046	0.52	AUC = 0.768
Number of sites with peritoneal disease	ALDH3A2	0.03	0.51	*τ* = 0.36
	CRIP2	0.01	0.29	*τ* = 0.439
Shape of peritoneal disease	MAGEA4	0.04	0.52	

### CT-based intra- and inter-site heterogeneity metrics were associated with an abundance of several proteins

The protein abundance of three proteins was associated with intra- and inter-site tumor heterogeneity texture metrics (Table 5). For example, cluster site entropy was positively correlated with the abundance of STXBP2 (*p* = 0.007, τ = 0.432) and negatively with ASS1 (*p* = 0.011, τ = − 0.364). Cluster standard deviation was positively correlated with an STXBP2 (*p* = 0.05, τ = 0.453). Cluster dissimilarity was positively correlated with STXBP2 (*p* = 0.03, τ = 0.368) and negatively with ASS1 (*p* = 0.009, τ = − 0.427) and CBK (*p* = 0.047, τ = − 0.326). However, none of these associations were significant after correction for multiple testing through the Benjamini-Hochberg procedure.

**Table 5:** Association between CT-based texture metrics and protein abundance in patients with HGSOC. Only associations for which the *p* value was less than 0.05 were included in this table. Positive values of *τ* indicate positive association between the texture metric and the level of expression of the protein whereas negative values of *τ* indicate negative association

Texture feature	Protein	*p* value	Corr. *p* value	Point estimate of metric of association
Cluster site entropy	ASS1	0.03	0.45	*τ* = − 0.364
	STXBP2	0.007	0.28	*τ* = 0.432
Cluster standard deviation	STXBP2	0.005	0.20	*τ* = 0.453
Cluster dissimilarity	ASS1	0.009	0.29	*τ* = − 0.427
	CKB	0.047	0.52	*τ* = − 0.326
	STXBP2	0.03	0.45	*τ* = 0.368

## Discussion

In this hypothesis-generating study, we investigated the relationship of CT imaging traits and CT-based texture measures of tumor heterogeneity to protein abundance in patients with HGSOC. This study is part of the efforts of the NCI initiative to combine proteomic data available from TCGA with patient-matched CT images collected as part of the TCIA effort in ovarian cancer. Our results provide preliminary evidence that suggests possible associations between imaging traits, CT-based texture measures of tumor heterogeneity, and the abundance of several proteins. These results are a step forward to the development of models that integrate clinical, proteomic, and radiomic data to predict meaningful clinical endpoints and facilitate tailored therapies.

Tumor involvement of the mesentery, which is a known important limiting factor in primary debulking surgery, was negatively correlated with the protein abundance of cysteine rich protein 2 (CRIP2). This correlation was even significant after correction for multiple testing. CRIP2 regulates cell proliferation, and acts as a tumor suppressor [33, 34]. In addition, the presence of supradiaphragmatic lymphadenopathy that causes patients to be up-staged to stage IV disease was positively associated with the protein abundance of MAGE family member A4 (MAGE4). Increased MAGE4 expression in ovarian cancer cells is an independent predictor for mortality in associated with worse overall survival [35].

This study also provides the first insights into possible associations between measures of CT-based tumor heterogeneity and protein abundance. The most interesting associations was with argininosuccinate synthase 1 (ASS1). HGSOC cells are known to express high levels of ASS1 [36, 37], and reduced levels of this protein correlate with platinum-based drug resistance in vitro and in vivo, and with worse prognosis [38]. We found that more heterogeneous tumors had lower ASS1 expression in with more homogenous tumors. This would support previous finding higher tumor heterogeneity measured by CT is predictive for worse survival [18].

A few studies, thus far, have applied radiomics in patients with ovarian cancer, mainly evaluating the ability of texture features to characterize tumor tissue [15, 17, 39, 40] and predict outcome [14, 15, 17, 18, 21, 22, 39]. Best to our knowledge, our work is the first that combines proteomic and radiomic data in patients with HGSOC, thereby facilitating the integration of multiple layers of data that is needed for the realization of precision medicine in oncological patients [20].

Our study was primarily limited by the retrospective design, the small study population, and the lack of a validation cohort. Due to the small sample size, there are inevitable biases in extracting CT textural features, with the risk of both false-positive and false-negative results. Therefore, we a priori selected only three texture metrics to assess IISTH, which had already been validated in larger studies to be predictive of treatment response and survival in patients with HGSOC [18, 19]. In addition, the process of selecting proteins may have screened out proteins that may be important. However, we think that this approach is justified, as the main aim of this study was to reduce the number of selected proteins to avoid false-positive correlations between protein expression and CT data. A larger study is needed for a more definitive analysis on the association between imaging traits and texture metric and CLOVAR subtypes, as well as from integrating the imaging, genomics, and proteomics data to predict response to treatment and outcome. However, since the TCGA/TCIA has been the only publicly available cohort of HGSOC patients with imaging, proteomics, and genomics data to date, this study was designed as a small hypothesis-generating study rather than a confirmative study.

In conclusion, this study provides the first insights into the potential associations between CT imaging traits and CT-based texture measures of tumor burden heterogeneity and the abundance of several tumor-associated proteins. Future, larger, prospective studies, such as that being conducted in the Applied Proteogenomic Organizational Learning and Outcomes (APOLLO) Research Network, are needed to validate our findings and enable the integration of clinical data, proteomics and radiomics to guide tailored therapies.

## Electronic supplementary material

ESM 1 Protein abundance of the 16 selected proteins (DOCX 17 kb)

## Abbreviations

AUC: Area under the receiver operating characteristic curve
CLOVAR: Classification of ovarian cancer transcriptomic profiles
CT: Computed tomography
CV: Coefficients of variation
HGSOC: High-grade serous ovarian cancer
HIPAA: Health Insurance Portability and Accountability Act
IISTH: Intra- and inter-site heterogeneity measures
MRI: Magnetic resonance imaging
PROVAR: Protein-driven index of ovarian carcinoma
TCGA: The Cancer Genome Atlas
TCIA: The Cancer Imaging Archive
VOI: Volume of interest
