# Supplementary material for: Integration of proteomics with CT-based qualitative and radiomic features in high-grade serous ovarian cancer patients: an exploratory analysis
Source: Eur Radiol. 2020 Apr 6;30(8):4306–16. doi: 10.1007/s00330-020-06755-3 (PMC7338824; doi:10.1007/s00330-020-06755-3)
Supplement: Supplementary file 1 — Protein abundance of the 16 selected proteins (DOCX 17 kb) [file 330_2020_6755_MOESM1_ESM.docx]

**Supplementary Materials**

| **Supplementary Table 1. 26 Proteins analysed** | | | |
| --- | --- | --- | --- |
| ALDH2 | ALDH3A2 | ALDH4A1 | ASS1 |
| CKB | CNDP2 | CRIP2 | GPI |
| HMGA2 | MAGEA4 | PLAA | REEP6 |
| SARS | SEC24B | STXBP2 | TK1 |
